# From shape to number: Shape-from-dots homogeneity boosts groupitizing enumeration

**DOI:** 10.3758/s13423-025-02755-w

**Published:** 2025-09-26

**Authors:** Andrea Adriano, Michaël Vande Velde

**Affiliations:** 1 Department of Psychology, Sapienza University of Rome, Rome, Italy; 2 Laboratoire Cognition Langage et Développement, Université Libre de Bruxelles, Brussels, Belgium

**Keywords:** Geometric regularity, Groupitizing, Gestalt perception, Numerosity, Shape processing

## Abstract

Enumerating a large set of objects (e.g., more than four items) can be accomplished more quickly and/or accurately when the objects are grouped into clusters based on Gestalt principles such as proximity and color similarity, a phenomenon known as “groupitizing.” However, whether other visuospatial features can similarly influence this mechanism remains unclear. This study investigated the impact of a novel feature: shape-from-dots homogeneity. Participants performed a simple enumeration task involving dot patterns, ranging from four to 20 items, spatially arranged in small clusters. In Experiment 1, the dots within the clusters were placed to form either *homogeneous* patterns of regular quadrilaterals (e.g., squares) or *heterogeneous* patterns of irregular, randomly shaped quadrilaterals. To test whether the effect was simply due to symmetry/canonicity, in Experiment 2, the dots within the clusters were placed to form either *homogeneous* patterns of regular quadrilaterals (e.g., squares) or *homogeneous* patterns of irregular, randomly shaped quadrilaterals. The results revealed that enumeration reaction times were significantly faster when clusters formed homogeneous shapes compared to heterogeneous ones (Experiment 1), while no difference was found when both patterns contained homogeneous arrays independently of the shapes (Experiment 2), ruling out that the effect was merely driven by spatial symmetry or canonicity. These findings indicate a close interaction between general shape processing and numerosity perception in the Groupitizing mechanism. This suggests that shape-from-dots homogeneity can facilitate numerosity processing akin to other Gestalt principles, likely promoting a multiplication mechanism.

**Supplementary Information:**

The online version contains supplementary material available at 10.3758/s13423-025-02755-w.

## Introduction

Human beings possess advanced mathematical abilities supported by specific neural circuits that enable efficient task-solving. Functional magnetic resonance imaging (fMRI) studies consistently highlight the role of bilateral frontal, intraparietal, and ventrolateral temporal regions in advanced mathematical processing (Amalric & Dehaene, 2016; Amalric et al., 2018), well separated from language-related networks in the ventral inferior-frontal and anterior-temporal cortex. However, evidence suggests that even higher mathematical knowledge may stem from rudimental cognitive mechanisms responsible for extracting and representing numerical information from the environment. The first key non-verbal cognitive ability is the subitizing mechanism (Mandler & Shebo, 1982; Revkin et al., 2008), which allows rapid and precise estimation of small set sizes (up to three to four items). For larger sets, the Approximate Number System (ANS; Dehaene et al., 1998) allows the processing of non-symbolic numerosity approximately, adhering to Weber’s law. Numerical tuning of the intraparietal sulcus has been observed during passive observation of dot arrays and through adaptation techniques (Piazza et al., 2004; see also Harvey et al., 2013), pointing to an ancient system shared with other species (e.g., Agrillo et al., 2009; Nieder & Miller, 2004) and active from early years of life (Brannon et al., 2004; Xu & Spelke, 2000). A third foundational ability is the spatial representation of numerical magnitude along a mental number line, as demonstrated by the spatial-numerical association response code (SNARC) effect (e.g., faster responses to small numbers with the left hand and to large numbers with the right hand; Dehaene et al., 1993) first observed in parity judgments tasks. This ability has also been found with dot arrays in human adults (Adriano et al., 2022; Nemeh et al., 2018) as well as in chicks (Rugani et al., 2015) and newborns (de Hevia et al., 2017), suggesting a biological origin of this effect.

A more recent skill, called “groupitizing” (Beckwith & Restle, 1966; Starkey & McCandliss, 2014), has been proposed. This mechanism involves chunking larger numerical sets (exceeding four items) into smaller, subitizable groups based on Gestalt principles like spatial proximity, color or shape similarity, thus enhancing enumeration accuracy and speed (e.g., Pan et al., 2021). Notably, Ciccione and Dehaene (2020) demonstrated a significant link between visual groupitizing and mathematical aptitude, suggesting its reliance on mental operations such as multiplication or combined multiplication and addition. Moreover, this relationship strengthens with education (Guillaume et al., 2023; Starkey & McCandliss, 2014). Recent studies provide further insights into groupitizing mechanisms. Maldonado Moscoso et al. (2022a, 2022b) reported that visual groupitizing activates the left angular gyrus – an area associated with multiplication (Cipolotti et al., 1991; Dehaene & Cohen, 1995; Gerstmann, 1940) – as well as bilateral parietal regions. Additionally, an EEG study by Caponi et al. (2023) revealed that the N1 peak latency is influenced more by the number of subgroups than the total number of items, suggesting that clustering impacts early visual processing stages.

Therefore, besides classic visual Gestalt cues such as proximity or color similarity, it is still unknown whether other visuospatial features can influence the groupitizing mechanisms. For example, numerosity perception within the subitizing range is closely linked to geometric cues involved in shaping an object’s form. This relationship has been demonstrated using dice dot patterns (Heine et al., 2011; Logan & Zbrodoff, 2003; Mandler & Shebo, 1982) and arrays with dots positioned at the corners of shapes such as triangles, squares, pentagons, and hexagons (Gheorghiu & Dering, 2020; Gheorghiu & Goldschmitt, 2022). These studies suggest that shape coding precedes numerosity estimation. Further evidence supporting this idea comes from studies indicating that sensitivity to visual forms, such as Glass patterns, can predict numerical abilities (Castaldi et al., 2022) and that visual form perception can mediate the relation between ANS skills and symbolic mathematical skills (Zhang et al., 2019). Furthermore, several studies suggest that symmetry affects ANS (Adriano & Ciccione, 2024; Apthorp & Bell, 2015; Maldonado Moscoso et al., 2022a, 2022b) as well as the subitizing mechanism (Hsin, Lo, & Tseng, 2021). These studies underscore the importance of visual shape processing in numerosity estimation within both the subitizing range and the ANS range (e.g., larger than 4 items). Furthermore, besides shape, recent works have reported that the arrays'visual coherence (e.g., homogeneity) consistently influences the perception of approximate numerosity. That is, variance in item orientation and color has a parametric effect on perceived numerosity, where more coherent arrays are perceived as having a larger numerical value compared to less coherent arrays (DeWind et al., 2020; Qu et al., 2022).

However, no study has investigated whether other visual features can influence the groupitizing mechanism. For instance, it has been reported that shape information can readily be derived from spatial dot patterns, even in non-numerical tasks (Baker & Kellman 2024). Here, we designed a psychophysical study to examine whether *shape-from-dots homogeneity* can influence groupitizing skills. We generated arrays of dots (from four to 20 items) containing dot clusters or chunks displayed according to two visual cues. In Experiment 1, in one condition, the clusters in the arrays were formed by *homogeneous* shapes, with dots forming regular shapes such as squares. In another condition, the clusters were formed by irregular random *heterogeneous* quadrilaterals. In Experiment 2, to disentangle whether the effect could be due to a mere shape-regularity advantage (e.g., symmetry and canonicity) or a genuine homogeneous-shape benefit, we presented *homogeneous* arrays of squares with *homogeneous* arrays of random irregular quadrilaterals. We asked participants to report the number of dots in each array using a simple forced-choice enumeration task. We predicted that arrays containing homogeneous shapes (formed by the spatial disposition of the dots) should promote multiplication skills, thus improving the performance compared to arrays with heterogeneous irregular shapes, as shown for other Gestalt cues (Experiment 1). Crucially, if the effect is merely due to a shape regularity/symmetry advantage, it should be present even in Experiment 2. On the contrary, no difference should be found between conditions if the effect is due to a true homogeneous-shape general visual benefit. To anticipate the results, in line with our prediction, we found that participants enumerated arrays with homogeneous geometric shapes faster than heterogeneous ones in Experiment 1. At the same time, no difference between conditions was found in Experiment 2. These results highlight the strict interactions between shape processing and groupitizing skills.

## Experiment 1: Homogeneous squares vs Heterogeneous irregular quadrilaterals

### Methods

#### Participants

To determine the sample size, we ran an a priori power analysis using G*Power 3.1 (Faul et al., 2009). Following the paper of Anobile et al. (2020), who found a partial eta-squared (η^2^_p_) for the effect of grouping over response times (RTs) of .23, we aimed at a significant within-factor effect (two levels), with a power of .80 and an alpha level of .05. To achieve this, a total of nine participants were required. The study included 16 participants (Mean Age = 29.125 years, SD = 5.6 years; 8 women, 8 men; 13 right-handed). Each participant signed an online informed consent document before the experiment began, and the study was conducted following the guidelines of the Declaration of Helsinki. The study was approved by the Local Ethics Committee.

#### Stimuli

**Fig. 1:**
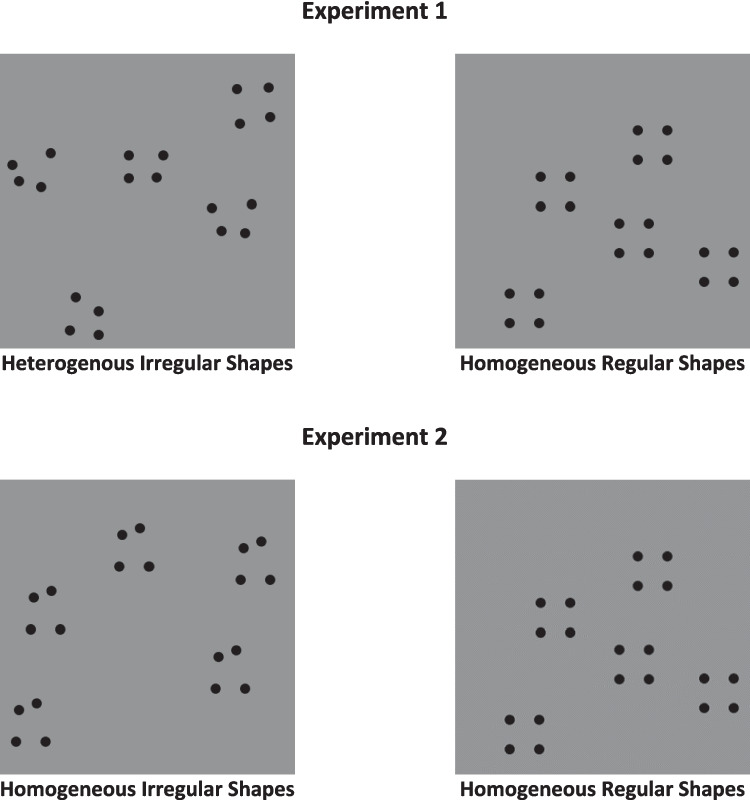
Stimuli used for the enumeration task in Experiment 1 (**top row**) and Experiment 2 (**bottom row**). In Experiment 1, each stimulus contained one, two, three, four, or five clusters of dots arranged to form *heterogeneous irregular* quadrilateral shapes (**left panel**) or *homogeneous regular* quadrilateral shapes (**right panel**). In Experiment 2, each stimulus contained one, two, three, four, or five clusters of dots arranged to form *homogeneous irregular* quadrilateral shapes (**left panel**) or *homogeneous regular* quadrilateral shapes (**right panel**). Both types of stimuli (irregular and regular) were generated in a similar way using a custom Python script. Regular Quadrilaterals were formed by four dots forming a square of 40 × 40 pixels, while irregular quadrilaterals were generated similarly, adding a jittering of ±10 pixels to each corner coordinates

The stimuli were randomly generated offline by a custom Python/Psychopy script and projected employing an online Psychopy routine (Peirce, 2007). The whole experimental set was composed of 250 test stimuli. Specifically, we generated a total of 125 stimuli (25 random spatial patterns × 5 numerosities) for the “Regular” shapes condition. Similarly, a total of 125 random spatial patterns were generated for the “Irregular” shapes condition. Each pattern could contain a total of 4, 8, 12, 16, or 20 black dots (diameter = 12 pixels; RGB = −1, −1, −1) spatially scattered in the pattern, thus forming one, two, three, four, or five clusters according to the condition. In the “Regular” shapes condition, each cluster was formed by four dots arranged to form a square of 40 × 40 pixels. In the “Irregular” shapes condition, each cluster was formed by four dots arranged to form a square initially, but a jittering of ± 10 pixels was then applied to the coordinates of each dot forming the corner of the shape, thus generating an irregular quadrilateral shape (Fig. 1). Furthermore, in both conditions, we constrained the single dots forming the clusters to be at least 60 pixels from the dots forming the other clusters in the pattern. The mean distance among the dots forming the shapes for the 25 patterns in the Irregular conditions was tested with an independent-sample t-test against the mean distance of Regular patterns for each numerosity. No significant difference was found across numerosities (all *p* > .05). All the single items were drawn on a middle-gray background (RGB = 0, 0, 0) and were projected within a virtual squared panel (400 × 400 pixels).

#### Procedure

**Fig. 2:**
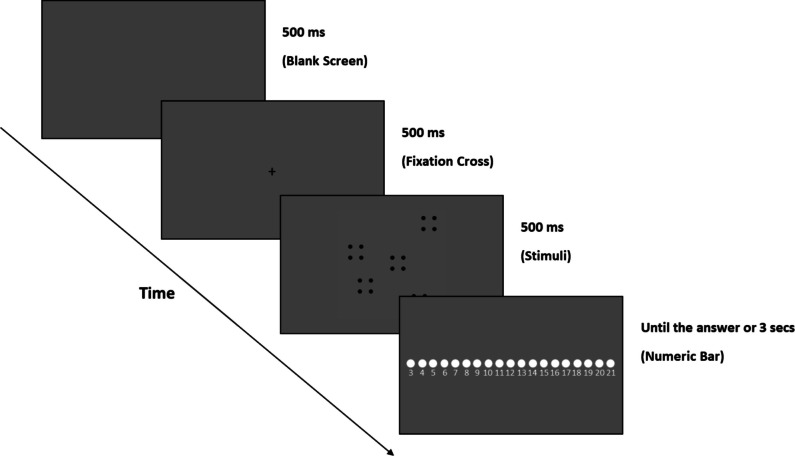
The forced-choice enumeration task. Each trial started with a gray background, followed by a fixation cross. Thus, the stimuli were projected. After the stimulus was offset, a numeric bar appeared on the screen, allowing the response

The stimuli were projected using an online Psychopy routine (Peirce, 2007), and all the experimental materials (stimuli, etc.) were downloaded before starting the experiment from the Pavlovia repository (www.pavlovia.org) to a temporary local folder stored on the computer of each participant. The general procedure was explained to each participant through detailed instructions provided on the display before starting the experiment. No information about the conditions was given to the subject. The participants performed a forced-choice enumeration task, asking them to judge the number of dots in each pattern. The experimental phase was preceded by a brief training of 20 trials (two trials for each of the five numerosities, for two shape conditions) without feedback to allow the subject to familiarize with the task. Each experimental trial started with a middle-gray background (RGB = 0, 0, 0) lasting 500 ms, followed by a black fixation cross (20 × 20 pixels; RGB = −1, −1, −1) projected for 500 ms, and then a pattern of dots appeared at the screen center for a further 500 ms (Fig. 2). After the stimuli offset, a numeric bar (800 × 40 pixels; RGB = 1, 1, 1) appeared in the screen center with a number from 3 to 21 as possible response choices. The numeric bar was presented on the screen until the participant’s answer or for a maximum of 3 s. The subjects performed their choice using the mouse pointer; hence, a new trial began. After the practice session, 250 experimental trials were thus presented (25 patterns × 5 numerosities × 2 shape conditions). The homogeneous and heterogeneous conditions were interleaved within the same experimental block to minimize potential order effects and to ensure that participants were equally engaged with all conditions throughout the task. The whole experiment lasted around 20 min.

#### Data analysis

The data were analyzed using the R-Studio (2018, v. 3.6.2; http://www.rstudio.com/) software package. Data for numerosity four and 20 were eliminated from the analysis to avoid anchoring effects to the extremes of the range. We measured the Accuracy and RTs of correct responses only as the primary dependent variables, as in Ciccione and Dehaene (2020). We measured numerical precision using the Weber fraction (Wf) as a secondary dependent variable. The Wf was calculated for each subject and condition (Halberda & Odic, 2014; Whalen et al., 1999) as the ratio between the standard deviation (SD) of the subjective estimates and the relative physical numerosity (N), as in Anobile et al. (2021). This secondary independent analysis was performed to verify overall adherence to Weber’s Law. Subjective estimates outside ± 2.5 SD from the mean for each condition, for each participant, were discarded from the analysis. When the sphericity assumption was violated, we applied the Greenhouse-Geisser epsilon (ε) correction and reported the original *F*, df, and corrected *p*-values.

### Results

**Fig. 3:**
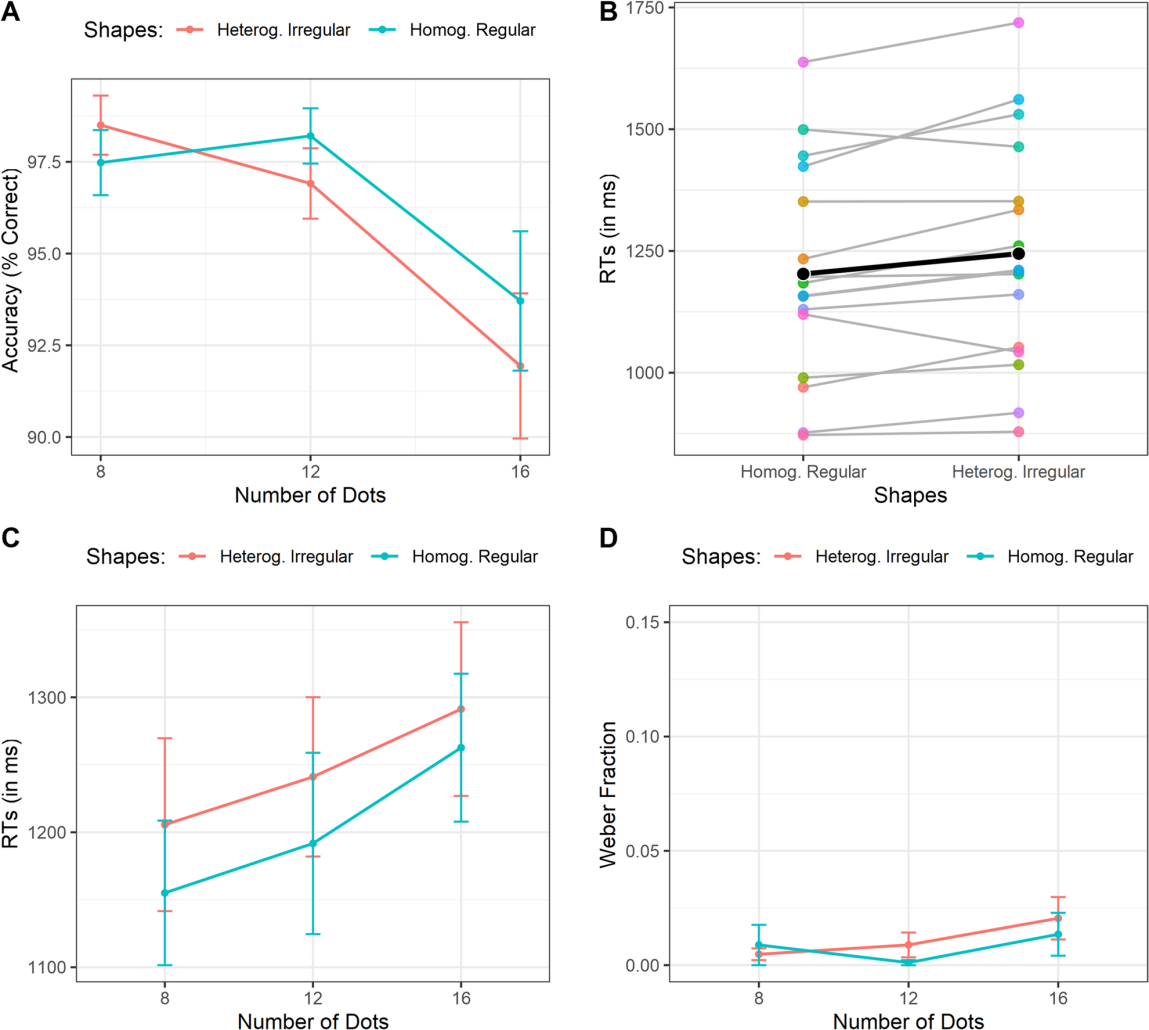
**(A)** Accuracy rate as a function of the number of dots and the shape condition. (**B)** Reaction times as a function of the shapes condition. The colored dots represent the individual mean of each subject. The black dot represents the group mean. (**C)** Reaction times as a function of the number of dots and the shape condition. (**D)** Weber fraction as a function of the number of dots and the shapes condition. Bars represent ± 1 standard error of the mean (SEM)

First, we performed a 2 × 3 repeated-measures ANOVA with the Shape condition (Homogeneous Regular vs. Heterogeneous Irregular) and the Number of Dots (8, 12, 16) as within factors and the accuracy as the dependent variable. The main effect of the within-variable Shape condition was not statistically significant, suggesting no difference in the accuracy between Homogeneous Regular and Heterogeneous Irregular shapes, *F*(1, 15) = 0.75, *p* = .39, η^2^_p_ = .048. Furthermore, the main effect of the within variable Number of Dots was significant, *F*(2, 30) = 10.587, ɛ = .68, *p* = .002, η^2^_p_ = .41. As expected, the accuracy decreased as the number of dots in the arrays increased. A polynomial trend analysis shows a significant decreasing linear trend, *t*(30) = −4.1, *p* < .001. Finally, the interaction between the two variables did not reach statistical significance, *F*(2, 30) = 2.134, *p* = .13, η^2^_p_ = .125 (Fig. 3A).

Hence, we performed a similar 2 × 3 repeated-measures ANOVA analysis over the RTs of correct response only. This time, as expected, we found a significant main effect of the Shape condition, *F*(1, 15) = 9.42, *p* = .008, η^2^_p_ = .38. As can be observed in Fig. 3B, we found that overall participants processed the arrays faster with Homogeneous Regular clusters compared to the Heterogeneous Irregular ones. We also found a significant main effect of the Number of Dots, *F*(2, 30) = 6.32, *p* = .005, η^2^_p_ = .29, suggesting that participants were slower in giving the answers when the number of dots increased. A polynomial trend analysis shows a significantly increasing linear trend, *t*(30) = 3.5, *p* < .001. Finally, the interaction between the Shape condition and the Number of Dots, *F*(2, 30) = 0.26, *p* = .77, η^2^_p_ = .017, was not statistically significant. However, as can be observed in Fig. 3C, the participants present a stronger homogeneous shape-from-dots benefit for numerosities up to 12 dots (three groups), and then the effect seems less intense. This is confirmed by planned contrasts (Homogeneous Regular vs. Heterogeneous Irregular) for each numerosity. Indeed, the paired contrast was significant for the numerosity 8, *t*(45) = 2.09, *p* = .042, and the numerosity 12,* t*(45) = 2.04, *p* = .047, but not for the numerosity 16, *t*(45) = 1.18, *p* = .23. This likely suggests that an upper limit in the number of groups/clusters that could be processed in parallel was reached (e.g., Halberda et al., 2006).

Finally, we analyzed the CoV using a similar 2 × 3 repeated measures ANOVA. We found that the effect of the Shape condition did not significantly affect the precision of estimation, *F*(1, 15) = 0.60, *p* = .45, η^2^_p_ = .039. Furthermore, the main effect of the Number of Dots, *F*(2, 30) = 1.8, *p* = .18, η^2^_p_ = .10, was not statistically significant, suggesting a constant precision over the numerosities as expected by Weber’s Law. Similarly, the interaction between the two variables did not reach statistical significance, *F*(2, 30) = 1.62, *p* = .21, η^2^_p_ = .097, Fig. 3D.

## Experiment 2: Homogeneous squares vs Homogeneous irregular-quadrilaterals.

### Methods

#### Participants

A new sample of 15 participants (Mean Age = 28.53 years, SD = 7.46 years; 9 women, 6 men, 10 right-handed) participated in the study. Each participant signed an online informed consent document before the experiment began, and the study was conducted following the Declaration of Helsinki. The study was approved by the Local Ethical Committee.

#### Stimuli, Procedure & Data Analysis

The stimuli were generated as in Experiment 1. The only difference was that we generated 25 random patterns for each target numerosity (4, 8, 12, 16, 20), always containing a *homogeneous* sample of random *irregular* quadrilaterals or a *homogeneous* sample of *regular* squares (as in Experiment 1; see Figs. 1 and 2). That is, while in the previous experiment the stimuli containing irregular shapes were composed of quadrilaterals of varying forms (e.g., heterogeneous irregular shapes), in the present experiment the stimuli contained random irregular quadrilaterals that were identical within each cluster (e.g., homogeneous irregular shapes). The procedure and the data analysis were performed as in the previous experiment.

### Results

**Figure 4:**
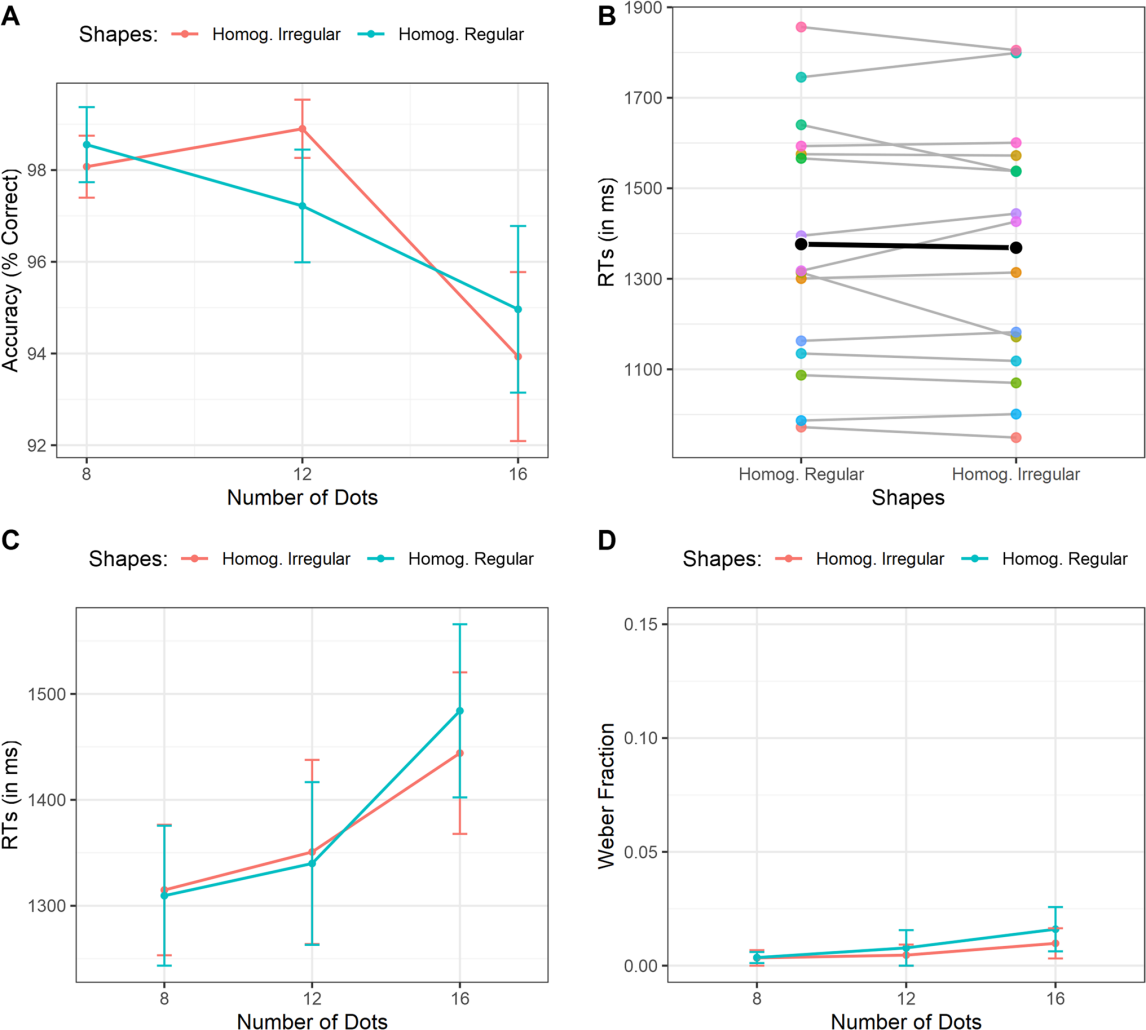
**A)** Accuracy rate as a function of the number of dots and the shape condition. **B)** Reaction times as a function of the shapes condition. The colored dots represent the individual mean of each subject. The black dot represents the group mean. **C)** Reaction times as a function of the number of dots and the shape condition. **D)** Weber fraction as a function of the number of dots and the shapes condition. Bars represent ± 1 SEM

We conducted a 2 × 3 repeated measures ANOVA with Shape condition (Homogeneous Regular vs. Homogeneous Irregular) and Number of Dots (8, 12, 16) as within-subject factors and accuracy as the dependent variable. The main effect of the Shape condition was not significant, indicating no accuracy difference between Homogeneous Regular and Homogeneous Irregular shapes, *F*(1, 14) = 0.011, *p* = .91, η^2^_p_ = .001. In contrast, the Number of Dots had a significant main effect, *F*(2, 28) = 5.9, ɛ = .65, *p* = .019, η^2^_p_ = .29, with accuracy decreasing as the number of dots increased. A polynomial trend analysis confirmed a significant linear decline, *t*(28) = −3.07, *p* = .005. The interaction between the Shape condition and the Number of Dots was not statistically significant, *F*(2, 28) = 1.03, *p* = .36, η^2^_p_ = .069, as shown in Fig. 4A.

We also ran a similar 2 × 3 repeated measures ANOVA on RTs for correct responses. This analysis revealed no significant main effect of Shape condition, *F*(1, 14) = 0.26, *p* = .61, η^2^_p_ = .019, with participants responding equally faster for arrays with Homogeneous Regular clusters and Homogeous Irregular ones (Fig. 4B). The Number of Dots also had a significant effect, *F*(2, 28) = 9.9, *p* < .001, η^2^_p_ = .41, with slower responses as dot quantity increased. A polynomial trend analysis showed a significant linear increase, *t*(28) = 4.23, *p* < .001. The interaction between the Shape condition and the Number of Dots was not significant, *F*(2, 28) = 0.97, *p* = .38, η^2^_p_ = .065, Fig. 4C. Hence, in line with our predictions, there was no particular difference between Shape conditions, suggesting that the first experiment's results were not merely driven by canonicity or symmetry.

Finally, we analyzed the coefficient of variance (CoV) using a 2 × 3 repeated-measures ANOVA. The Shape condition did not significantly influence the precision, *F*(1, 14) = 0.35, *p* = .56, η^2^_p_ = .024. Similarly, the Number of Dots had no significant effect, *F*(2, 28) = 1.71, *p* = .19, η^2^_p_ = .109, indicating stable precision across numerosities in line with Weber’s Law. The interaction between the two variables was also not significant, *F*(2, 28) = 0.116, *p* = .89, η^2^_p_ = .008 (Fig. 4D).

### Discussion

This study investigated a novel visual feature that influences the groupitizing mechanism: shape-from-dots homogeneity (Baker & Kellman 2024; Feldman, 1997). Specifically, we observed that arrays containing clusters of dots arranged in *homogeneous* regular geometric shapes, such as squares, are processed faster than arrays with clusters forming *heterogeneous* irregular quadrilaterals, as we found in Experiment 1. Furthermore, in Experiment 2, we ruled out that the effect was merely driven by spatial symmetry/canonicity in the subitizing range (e.g., squares are more symmetrical, see Hsin et al., 2021) or because squares are more frequent shapes since we found no difference in RTs when *homogeneous* regular quadrilaterals were tested with *homogeneous* irregular random quadrilaterals. Because there were no differences in accuracy or Weber fraction between conditions, the observed RTs effect cannot be attributed to changes in perceptual sensitivity, but instead point to other underlying mechanisms. Hence, the effect found in the first experiment is likely due to a genuine shape-from-dots homogeneity speed advantage (e.g., Baker & Kellman 2024). This effect underscores a strong relationship between shape processing and numerical perception through the groupitizing mechanism.

But how can this effect be explained? One promising interpretation is that dots arranged to form a small local cluster activate the perception of geometric shapes in the brain. Psychophysical evidence supports the notion that shape information can be readily extracted from spatial dot arrangements (Baker & Kellman, 2024; Feldman, 1997). In line with the multiplication hypothesis proposed by Ciccione and Dehaene (2020) to explain the groupitizing mechanism, when all the chunks share the same *homogeneous* shape (e.g., a quadrilateral), it is almost instantly visually evident that each chunk also shares the same numerosity (e.g., four items), thus the total number of items in the set can be immediately obtained by multiplying the number of vertices (e.g., four) by the number of sub-groups, hence increasing the speed of processing. Of course, when the shapes in the patterns are not homogeneous, it is less evident that each chunk contains the same number of dots since each shape should be scanned serially to check the vertex’s disposition (and thus the number of dots). Therefore, more time is needed to obtain the total number of dots. The effect we identified mimics the influence of classic Gestalt principles – such as color/shape similarity and proximity – previously reported in groupitizing studies, and further sustains the multiplication hypothesis (Ciccione & Dehaene, 2020). In both Experiment 1 and Experiment 2, arrays of 8, 12, and 16 dots were always organized into clusters of four items, so participants could immediately estimate two, three, or four sub-groups in each array, likely using the subitizing mechanism (Wege et al., 2022). However, in Experiment 1, the homogeneous‐regular condition presented clusters as perfect squares, allowing observers to infer “four dots per cluster” almost instantly from the canonical shape, whereas the heterogeneous‐irregular condition displayed each cluster as a different jittered quadrilateral, forcing participants to inspect each cluster individually to verify that it contained exactly four dots. Consequently, the reduced accuracy in that heterogeneous condition cannot arise from confusion about cluster count but rather from the increased perceptual demands of confirming cardinality across differently shaped clusters. In Experiment 2, both conditions – homogeneous‐regular and homogeneous‐irregular – used a single repeated shape for all clusters, so participants could immediately infer that every cluster contained four dots by recognizing one template, regardless of whether it was a square or an irregular quadrilateral; as a result, there was no accuracy or speed penalty when all clusters shared the same irregular form. Thus, the data indicate that cross‐cluster heterogeneity, not cluster number per se, impairs numerosity estimation by making it more difficult to verify “four dots per cluster.”

Therefore, as we found in Experiment 1, when multiple sub-groups are formed, the effect of shape homogeneity impacts the numerosity processing up to three chunks. Beyond this limit, the influence of shape homogeneity diminishes or becomes negligible, likely due to an upper bound on the number of groups that can be processed simultaneously (Halberda et al., 2006).

Overall, these results align with earlier studies demonstrating that shape processing (e.g., Gheorghiu & Dering, 2020; Gheorghiu & Goldschmitt, 2022) and homogeneity (DeWind et al., 2020; Qu et al., 2022) can influence numerical perception both within the subitizing range and the ANS range. Additionally, numerous studies suggest that shape regularities in stimuli are leveraged whenever available to achieve efficient processing and compression of information in working memory, thereby improving the handling of incoming information and facilitating memorization, anticipation, and outlier detection (e.g., Adriano et al. 2025; Al Roumi et al., 2023; Amalric et al., 2017; Biederman, 1987; Feldman, 1997, 2000; Sablé-Meyer et al., 2021). Hence, shape-from-dots homogeneity may represent another regularity that can be exploited to accelerate the enumeration process through multiplicative mechanisms (Ciccione & Dehaene, 2020). Future research should investigate several open questions to further elucidate the mechanisms underlying the “geometrizing” effect (e.g., the interaction between groupitizing and geometric cues) and its implications. For instance, future work could extend the current work by eliciting separate reports of cluster count versus dots‐per‐cluster. A follow‐up experiment, for example, could ask participants to first report “how many clusters” they see and then, on a separate screen, to report “how many dots” are in one randomly highlighted cluster. We predict that in the heterogeneous condition of Experiment 1, participants would be slower or less accurate when reporting “four” on that second step, compared to any homogeneous condition (regular or irregular). Moreover, employing eye‐tracking should be useful to measure inspection patterns and/or using ERP (event-related potential) to quantify the extra processing required when cluster shapes differ. In particular, eye‐tracking could reveal that in the *heterogeneous* condition of Experiment 1, observers make more fixations per cluster (or revisit clusters) to confirm dot count, whereas in any homogeneous condition, they require fewer fixations because the shared template suffices.

Similarly, future studies should study more geometric forms to identify which gives an advantage over irregular ones and between regular ones (e.g., hexagons vs. squares). Another promising direction involves exploring the neural correlates of this phenomenon through neuroimaging techniques such as fMRI or EEG. For instance, studies could aim to identify whether specific areas of the lateral occipital cortex (LOC) or ventral stream (e.g., Amir, Biederman, & Hayworth, 2011; Biederman, 1987; Kayaert et al., 2005; Kim & Biederman, 2012) are simultaneously involved in shape processing and numerical perception. Indeed, although the role of shape processing on numerosity processing is still largely unknown at the neural level, several pieces of evidence suggest that some areas might be good candidates for achieving a number-shape combination. For example, numerosity-tuned neurons have been localized through fMRI in the ventral stream of the cortex (Karami et al., 2023) near areas that have been classically recognized as areas supporting shape processing (Grill-Spector et al., 2001). Furthermore, as we reported above, arrays with symmetric disposition tend to be underestimated compared to non-symmetric arrays, suggesting that global shape processing is intertwined with numerosity processing (Adriano & Ciccione, 2024; Apthorp & Bell, 2015; Maldonado Moscoso et al., 2022a, 2022b; see also Hsin et al., 2021, for an effect of symmetry in the subitizing range), suggesting the implication of a region of the lateral occipital complex (LOC) dedicated to symmetry processing and object segmentation (for a review, see Bertamini et al., 2018). Also, visual form perception has been suggested as the mediating factor between ANS skills and advanced mathematics (Zhang et al., 2019). Another avenue for exploration is the developmental trajectory of the geometrizing effect. Longitudinal studies with children could examine how sensitivity to geometric regularities interacts with the maturation of numerical cognition. Such research might reveal whether exposure to structured visual environments, such as games or educational tools emphasizing geometric regularities, can enhance numerical processing skills.

## Conclusions

In this study, we introduced and examined a novel phenomenon termed “geometrizing,” which highlights the influence of geometric cues on the groupitizing mechanism and numerical perception. Our findings demonstrate that the geometric arrangement of homogeneous shapes promotes more efficient numerical processing compared to heterogeneous shape configurations. This effect likely arises from the interplay between shape recognition and numerical processes, leveraging the brain’s sensitivity to geometric properties and regularities.

Future research should build on these insights by investigating the neural and developmental underpinnings of the geometrizing effect. Exploring its presence in other species and potential applications in education or design could provide valuable perspectives. Understanding how geometric and numerical information are integrated at both behavioral and neural levels will deepen our knowledge of the mechanisms that support human perception, cognition, and learning.

## Supplementary Information

Below is the link to the electronic supplementary material.

Supplementary file1 (CSV 1 KB)

Supplementary file2 (CSV 1 KB)

Supplementary file3 (CSV 1 KB)

Supplementary file4 (CSV 1 KB)

Supplementary file5 (CSV 1 KB)

Supplementary file6 (CSV 1 KB)

## Data Availability

The datasets generated during the current study are available online as Supplementary Materials.

## Code availability

The code for the analysis is available online as Supplementary Materials.

## Declarations

### Open practices statement

The data for all experiments are available online as Supplementary Materials.
